# Simple liquid chromatography-electrospray ionization ion trap mass spectrometry method for the quantification of galacto-oxylipin arabidopsides in plant samples

**DOI:** 10.1038/s41598-020-68757-x

**Published:** 2020-07-20

**Authors:** Manon Genva, Mats X. Andersson, Marie-Laure Fauconnier

**Affiliations:** 1 Laboratory of Chemistry of Natural Molecules, Gembloux Agro‐Bio Tech, University of Liège, Passage des Déportés 2, 5030 Gembloux, Belgium; 2 Department of Biological and Environmental Sciences, University of Gothenburg, Box 461, 405 30 Göteborg, Sweden

**Keywords:** Mass spectrometry, Plant immunity, Plant stress responses

## Abstract

A simple and sensitive method to quantify five different arabidopsides by HPLC—ion trap mass spectrometry in complex plant samples was developed and validated. Arabidopsides are oxidized galactolipids first described in *Arabidopsis thaliana* but also produced by other plant species under stress conditions. External calibration was performed using arabidopsides purified from freeze-thawed *Arabidopsis* leaves. Lipids were extracted and pre-purified on an SPE silica column before HPLC–MS analysis. Arabidopsides were separated on a C18 column using a gradient of mQ water and acetonitrile:mQ water (85:15) supplemented with formic acid (0.2%) and ammonium formate (12 mM). The method was validated according to European commission decision 2002/657/CE. LOD, LOQ, linearity, intra-day and inter-day precision and accuracy, selectivity, matrix effects and recoveries were determined for the five metabolites. The established method is highly selective in a complex plant matrix. LOD and LOQ were, respectively, in the range 0.098–0.78 and 0.64–1.56 µM, allowing the arabidopside quantification from 25.6–62.4 nmol/g fresh weight. Calibration curve correlation coefficients were higher than 0.997. Matrix effects ranged from -2.09% to 6.10% and recoveries between 70.7% and 109%. The method was successfully applied to complex plant matrixes: *Arabidopsis thaliana* and *Nasturtium officinale*.

## Introduction

Oxylipins are structurally diverse plant metabolites produced following the oxidation of unsaturated fatty acids and include aldehydes, divinyl ethers, oxo-, keto-, hydroxyl- and hydroperoxy acids^1–3^. These molecules play crucial roles in plants, as they are involved in developmental processes and defence responses^4^. Among the different families of plant oxylipins, jasmonates comprise all molecules formed after α-linolenic and hexadecatrienoic acids enzymatic transformation into jasmonic acid and its wide range of derivatives. Indeed, jasmonic acid can be converted by many metabolic pathways into different active, inactive or partially active compounds, showing the high complexity of the jasmonate pathways. Those ubiquitous plant metabolites have been studied for many years, as both jasmonic acid derivatives and some of its precursors modulate plant gene expression, leading in crucial modifications in plant developmental, physiological and defence processes^5^. As examples, jasmonic acid derivatives and its precursors are directly implicated in flower development, leaf senescence, seed maturation, the attraction of insects for pollination and defence against herbivores^3–5^. Besides functions as signals in planta, in vitro experiments showed high antimicrobial activities of various oxylipins against diverse pathogens: bacteria, fungi and oomycetes^6–9^. These experiments have also shown that different oxylipins and their stereoisomers have distinct biological activities against pathogens^4^. Moreover, it was recently reported that some oxylipin-like fatty acid hydroperoxides interact with plant plasma membrane lipids in vitro and can modify the plasma membrane organization^10^. All these reported insights highlight the crucial functions of plant oxylipins in defence responses and the likely role of these compounds in plant–pathogen interactions^4^.

**Figure 1:**
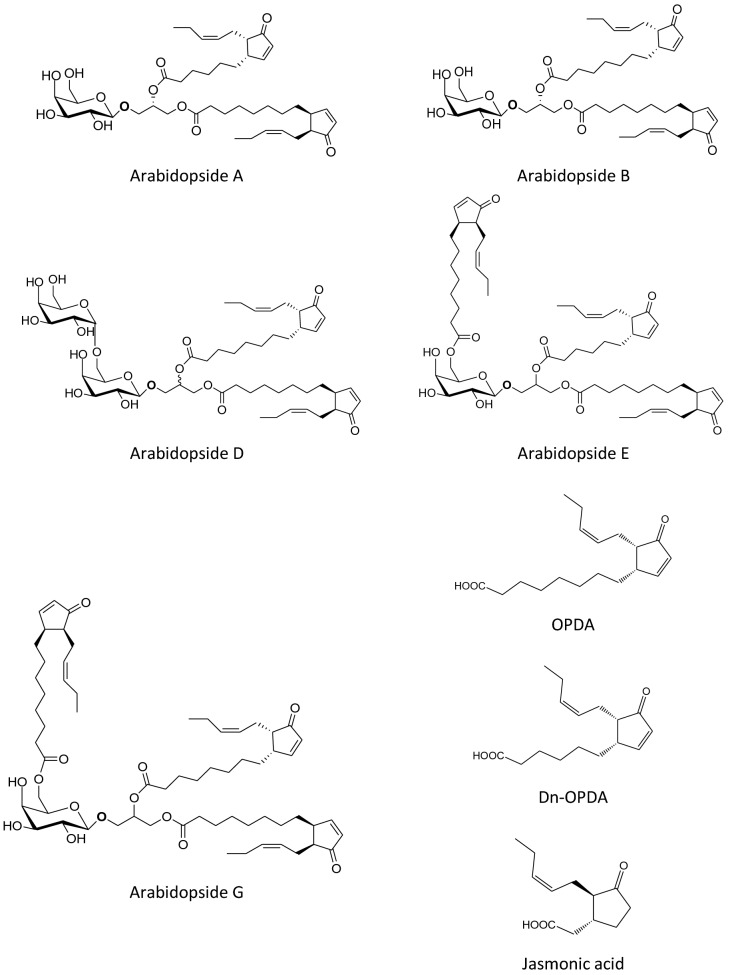
Structure of the five major arabidopside species (A, B, D, E and G), OPDA, dn-OPDA and jasmonic acid. Adapted from Genva, M. et al. (2019)^3^ with authorization. Arabidopsides A and B are (dn)OPDA containing MGDGs, arabidopside D is an OPDA containing DGDG, arabidopsides E and G are head group acylated derivatives of these.

While free oxylipins have been well studied and characterized, the biological properties of esterified glycerolipids like arabidopsides are still understudied. These oxylipins are produced following the enzymatic oxidation of chloroplast monogalactosyldiacylglycerols (MGDG) and digalactosyldiacylglycerols (DGDG), under a wide range of stress conditions (mechanical wounding, bacterial infection, low-temperature treatment etc.)^6,9,11–14^. Arabidopsides were initially described in *Arabidopsis thaliana* (L.) Heynh (hereafter *Arabidopsis*)^15,16^ but were subsequently also found in other plant species and families^17–22^. A wide range of different arabidopsides has now been described, all of them containing at least one galactose and one esterified 12-*oxo*-phytodienoic acid (OPDA) or its 16C analogue, 12-dinor-*oxo*-phytodienoic acid (dn-OPDA). Free OPDA and dn-OPDA are both involved in jasmonic acid synthesis. Some typical arabidopside structures^3^ are gathered in Fig. 1. Arabidopsides A, B, D, E and G are the major arabidopside molecules found in plant species. Other arabidopsides, such as arabidopside C, have also already been found in some plant species, but in much lower quantities^11^.

The true function of arabidopsides in planta remains unclear, but it has been suggested that these compounds may play important roles in defence responses and developmental processes. Some arabidopsides can induce growth inhibition of cress roots^16,23^, and arabidopsides can accumulate in high proportions (up to ± 10% of total esterified fatty acids) under stress conditions^6,9,11,12^. However, it is not known whether or not arabidopsides play a direct role or if they are a source of free (dn)OPDA. The latter proposition is supported by the finding that arabidopsides can be a substrate for acyl lipases, allowing the release of free OPDA^24,25^. Released (dn)OPDA could be used for jasmonic acid synthesis but could also directly modulate gene expression as free OPDA is directly able to activate jasmonic acid-independent responses involved in the inhibition of seed germination^26^ and drought stress responses^27^, for instance. Moreover, in vitro studies have shown that some arabidopsides have antibacterial and antifungal properties^6,9,19^ indicating a direct biological effect. Arabidopsides are also able to induce plant senescence, which highlights a possible direct role of these molecules in plant developmental processes^3^. To better understand the functions of those molecules, the main difficulty is to be able to quantify precisely arabidopsides in plant tissues as low concentration modifications may induce crucial physiological changes.

As arabidopsides are not commercially available as pure standards, these compounds need to be first extracted and purified from plants, which makes their study challenging. Moreover, analytical methods did not allow direct analysis of esterified oxylipins until the last two decades. Usually, the fatty acid moieties of complex lipids are first released by hydrolysis before analysis. The main disadvantage of this technique is that the whole esterified oxylipin structure cannot be elucidated, as only the oxylipin part of the molecule is analysed^28,29^. Contemporary analysis methods for arabidopsides utilize HPLC–MS–MS^17,30,31^ or direct infusion MS–MS^11,12^ to quantify these molecules in plant samples. However, most of those methods use internal calibration with non-oxidized galactolipids^6,32^ which structurally differ from arabidopsides and might display very different analytical responses. There is thus a significant need in the development of analytical methods allowing precise quantifications of those molecules with important roles in plant defence and development. In this study, we report the first HPLC—ion trap mass spectrometry (HPLC-IT-MS) validated method for the determination of the five major arabidopsides using external calibration with highly purified arabidopside standards. Plant sample preparation was straightforward, as a simple SPE procedure was applied. An ion trap mass analyser was used for arabidopside quantification, as it allows the quantification of low abundance ions that are trapped and concentrated in the mass spectrometer before detection, allowing the quantification of low molecule levels^19,32,33^. The established method was successfully applied to the quantification of arabidopsides in *Arabidopsis* and *Nasturtium officinale* R. Brown.

## Results and discussion

### Arabidopside purification

**Figure 2:**
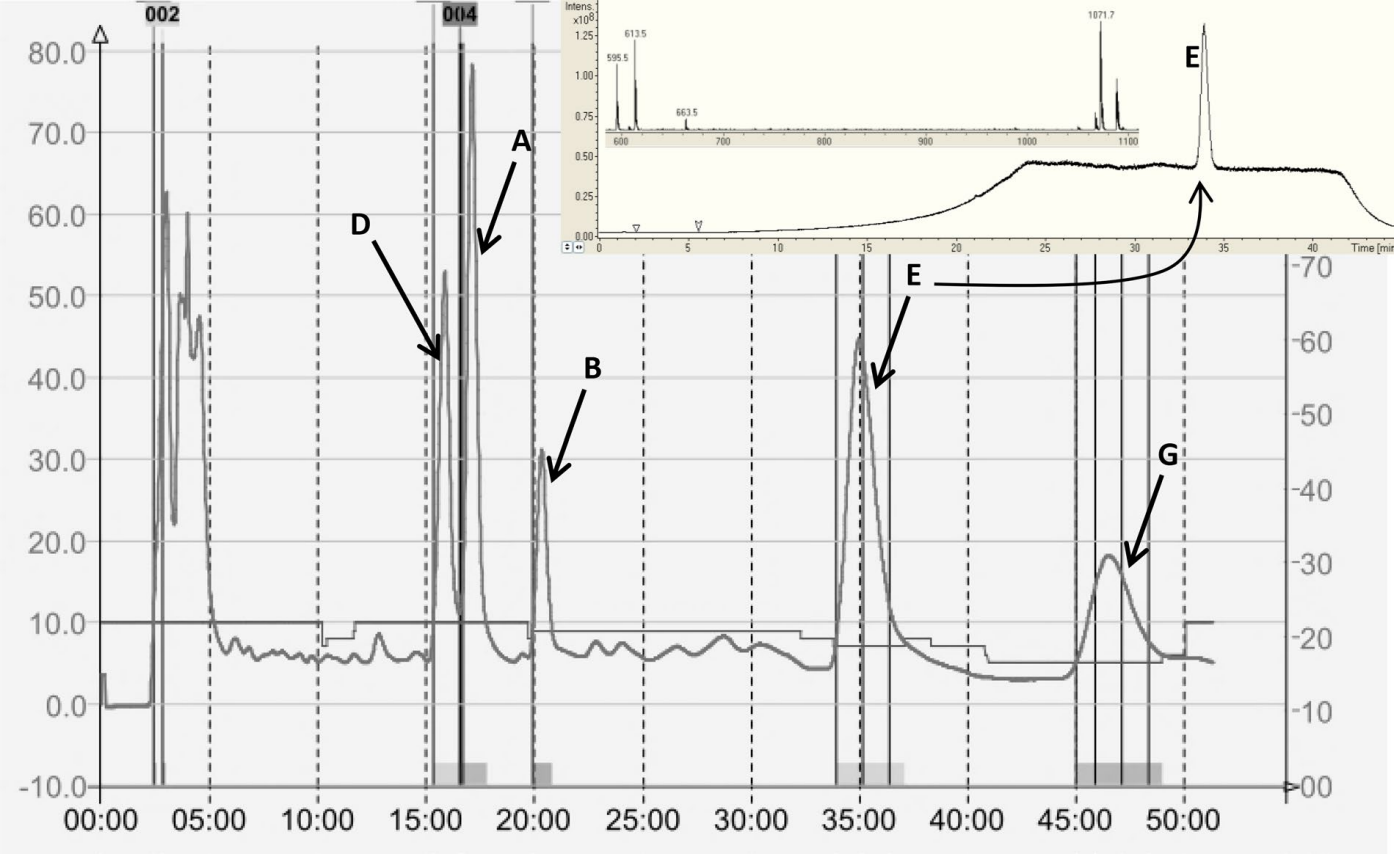
Preparative HPLC chromatogram obtained during arabidopside purification with UV detection at 220 nm. All arabidopsides were then analysed by HPLC–MS. Insert shows HPLC–MS TIC of arabidopside E and spectrum of the main peak. See Supplemental Fig. 1 for HPLC–MS TIC of all arabidopside standards.

As arabidopsides are as yet not commercially available, the five major arabidopsides were extracted and purified from freeze-thawed *Arabidopsis* leaves*.* After sample clean-up on a silica column (see the “Experimental” section), preparative-HPLC was carried-out. As shown in Fig. 2, the optimized gradient allowed a performant separation of all five arabidopsides, which were then analysed by HPLC–MS to confirm their identity (Supplemental Fig. 1). The identities were also confirmed by 2D-NMR (HSQC, COSY and HMBC), UV–visible and infrared spectroscopy (see all spectra for arabidopside A in supplementary data, supplemental Figs. 2–6). Arabidopside purities were evaluated from HPLC–MS-total ion chromatograms (Supplemental Fig. 1). HPLC–MS results showed that the five arabidopsides were recovered with the following purities: 99.4% for arabidopside A, 95.7% for arabidopside B, 92.4% for arabidopside D, 99.5% for arabidopside E and 90.8% for arabidopside G.

### Method development

**Figure 3:**
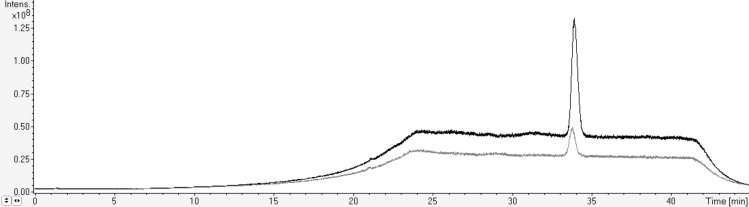
Total ion chromatogram (TIC) of arabidopside E detected with ion trap parameters optimized for oxidized acyl-MGDG detection (in black) and oxidized MGDG detection (in grey).

A method was developed to separate, detect and quantify arabidopsides in complex plant samples. A gradient of acetonitrile and water was chosen, giving the best peak resolution for elution of arabidopsides A, B, D, E and G with high specificity as no peaks in complex plant samples were eluted at the same arabidopside retention times. Ammonium formate and formic acid were added to the solvents to enhance electrospray ionization in positive mode. Results also highlighted that each class of arabidopsides needs specifically optimized parameters for its enhanced detection (Fig. 3). Ion trap parameters were then optimized for each different arabidopside species by direct infusion of the different molecules. The optimal parameters are summarized in Supplemental Table 1. The HPLC–IT–MS identification of each molecule was performed based on its retention time, the mass of its chosen adduct and the mass of a selected confirmation ion. The latter ones corresponded to the loss of one (for arabidopsides A and B) or two (for arabidopside D) sugar moieties and of one sugar moiety and one OPDA acyl-chain for arabidopsides E and G. For all arabidopsides, the selected confirmation ions were the same as those previously described^34^.

**Table 1:** Analytical method validation performance for the quantification of arabidopsides A, B, D, E and G by HPLC-IT-MS.

	Arabidopside A	Arabidopside B	Arabidopside D	Arabidopside E	Arabidopside G
Quantification ion	792.5 [M + NH_4_]^+^	820.5 [M + NH_4_]^+^	987.5 [M + Na]^+^	1,071.6 [M + Na]^+^	1,099.7 [M + Na]^+^
Confirmation ion	613.4 [M-C_6_H_10_O_5_]^+^	641.4 [M-C_6_H_10_O_5_]^+^	641.4 [M-2 C_6_H_10_O_5_]^+^	613.4 [M-C_6_H_10_O_5_–C_18_H_26_O_2_]^+^	641.4 [M-C_6_H_10_O_5_–C_18_H_26_O_2_]^+^
Limit of detection (µM)	0.098	0.20	0.39	0.78	0.29
Limit of detection (nmol/g fresh weight)	3.92	8.00	15.60	31.20	11.60
Limit of quantification (µM)	0.78	0.78	0.78	1.56	0.64
Limit of quantification (nmol/g fresh weight)	31.20	31.20	31.20	62.40	25.60
Calibration range (µM)	0.78–12.5	0.78–50	0 .78–12.5	1.56–50	0.64–24.8
Calibration equation	y = 5,141,152x − 494,739	y = 3,302,949x − 172,260	y = 1,866,658x − 81,263	y = 114,109x^2^ + 5,342,367x − 4,056,416	y = 476,900x^2^ + 14,824,447x
Coefficient of determination (R^2^)	0.9994	0.9992	0.9999	1.0000	0.9971
Intra-day precision Intra-day accuracy (%)	%RSD_r_ < 8.37 97.5–100	%RSD_r_ < 7.09 94.9–98.2	%RSD_r_ < 4.25 101–105	%RSD_r_ < 5.00 97.3–106	%RSD_r_ < 6.55 93.9–102
Inter-day precision Inter-day accuracy (%)	%RSD_R_ < 4.35 94.5–97.8	%RSD_R_ < 2.87 93.2–97.7	%RSD_R_ < 5.19 106–108	%RSD_R_ < 4.45 93.3–106	%RSD_R_ < 7.59 95.1–102
Matrix effect (%)	− 0.719 to 4.48	− 2.09 to 0.831	1.88–4.30	1.80–4.62	2.58–6.10
Recovery (%)	81.5	109	70.7	108	89.4

**Figure 4:**
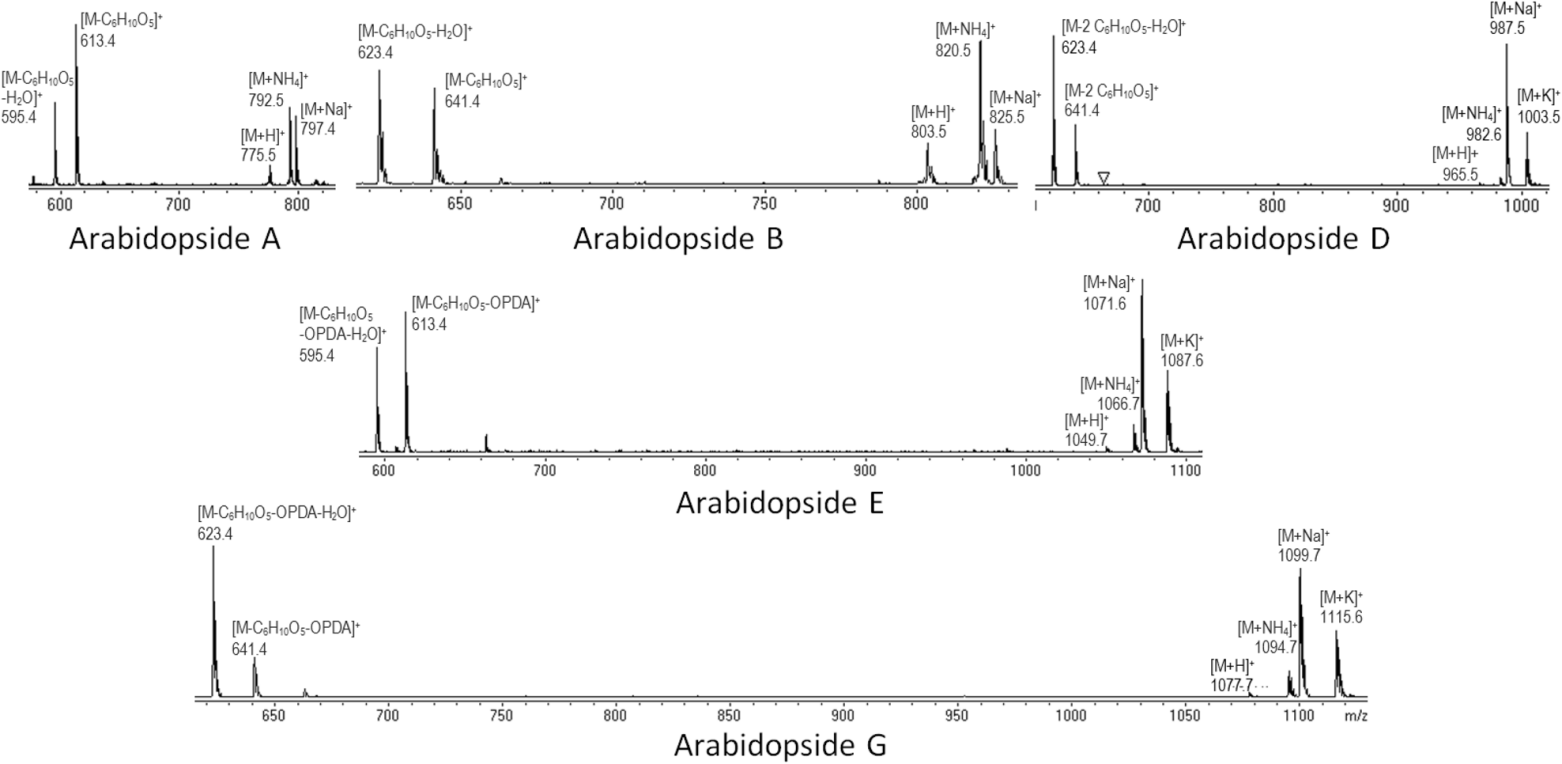
Major adducts and fragments during HPLC–MS analysis of arabidopsides A, B, D, E and G. for arabidopsides A and B, ammonium adducts were the most abundant and were then chosen for their quantification. For arabidopsides D, E and G, the sodium adducts that dominated were used for the quantification of those species. For all arabidopsides, the major detected fragments corresponded to sugar losses with or without the loss of an additional water molecule.

The area of a selected molecular ion adduct was used for each arabidopside quantification. The ratios between the different arabidopside adducts were compared for standard molecules and arabidopsides in extracted plant samples. Those ratios were constants in both standard molecules and complex plant samples, allowing the choice of the major adduct for each arabidopside molecule to increase the method sensitivity. The areas of the arabidopside A and B ammonium (M + 18 amu) adduct peaks were used for their quantification. For arabidopsides D, E and G, sodium adducts that dominated the mass spectrum (Fig. 4) were chosen for quantification purposes. Additionally, arabidopside G was eluted during a decrease in acetonitrile content where the baseline was not flat. Quantification of this molecule based on its adduct allowed enhanced precision in peak area determination.

### Validation of the established method

External calibration with authentic standards purified from disrupted plants was used for arabidopsides quantification. For arabidopsides A, B and D, linear calibration was observed whereas arabidopsides E and G required a second-degree polynomial calibration curve. Each calibration curve was the mean of three distinct curves and each point consisted of triplicate injections. For all studied arabidopsides, the coefficient of determination (R^2^) was higher than 0.997, showing that the data fitted well with the suggested regression models over the calibration range. The LOD was between 0.098 and 0.78 µM, while LOQ was from 0.64 to 1.56 µM for all tested analytes (Table 1). As this method was performed from approximatively 0.4 g of fresh plant material, it allowed the quantification of arabidopsides in plant samples from 25.6–62.4 nmol/g fresh weight.

**Figure 5:**
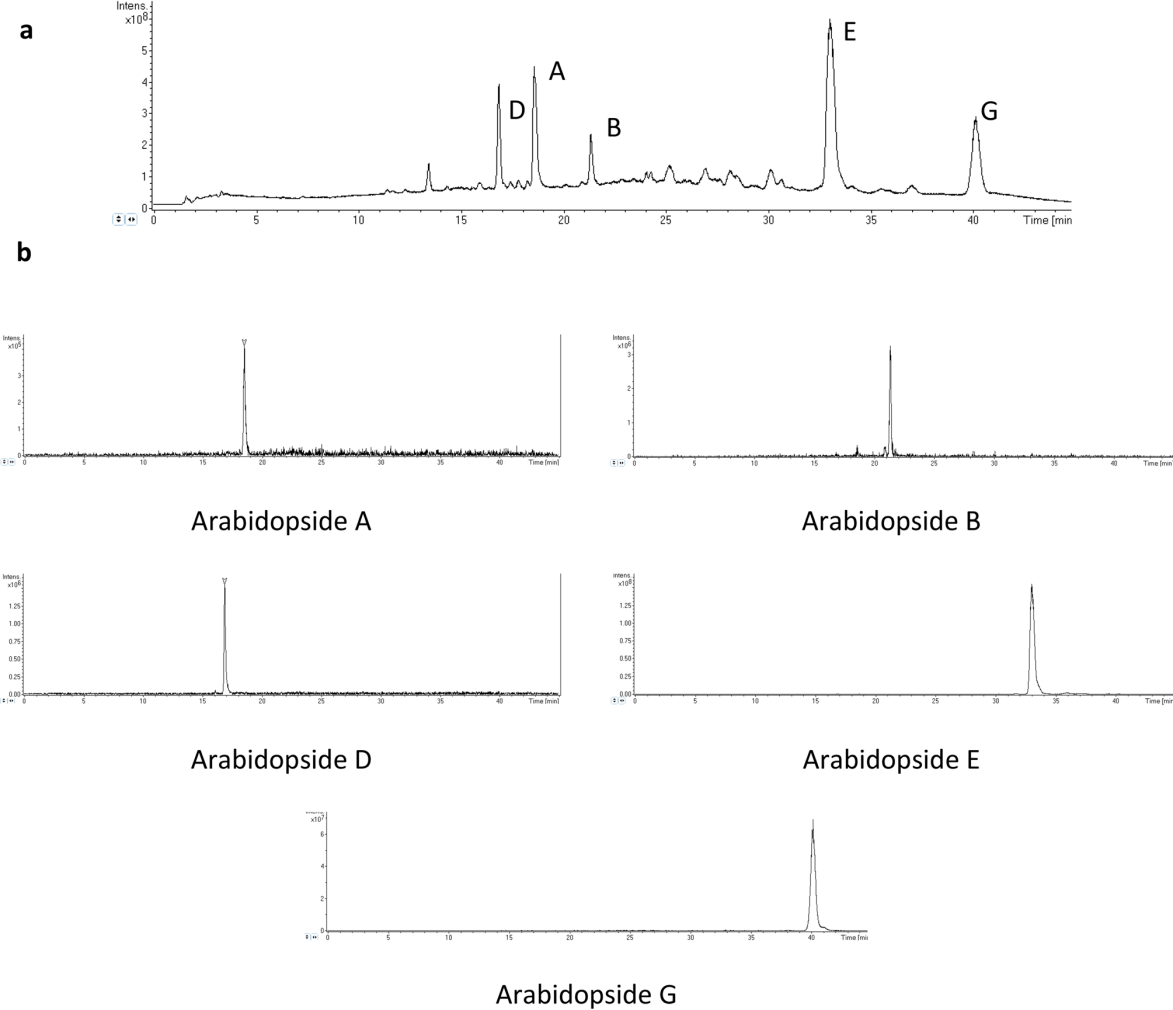
**(a)** Representative TIC of *A. thaliana* sample containing arabidopsides analysed with the method optimized for acyl-MGDG species. **(b)** Representative extracted ion chromatograms of arabidopsides A, B, D, E and G.

The specificity of the established method was evaluated by verifying the absence of interfering peaks coming from plant samples. Representative total ion chromatograms and extracted ion chromatograms of plant sample containing arabidopsides are shown in Fig. 5, and they illustrate the good specificity of the established method as no interfering peak was found for all five arabidopsides.

Intra-day (n = 6) and inter-day (n = 3) variations were tested to define the precision and accuracy of the method (Table 2). The established method showed low relative standard deviation (RSD) and high accuracy for all studied analytes with intra-day relative standard deviation percentages (%RSD_r_) between 4.25 and 8.37% and inter-day relative standard deviation percentages (%RSD_R_) between 2.87 and 7.59%. Intra-day and inter-day accuracy were, respectively 93.9–106% and 93.2–108%, showing the high precision and accuracy of the established method.

**Table 2:** The precision of the method. The precision of the developed method was evaluated by spiking blank samples with three concentrations of each arabidopside: LOQ, 1,5*LOQ and 2LOQ.

		Arabidopside A			Arabidopside B			Arabidopside D			Arabidopside E			Arabidopside G		
Added concentration (µM)		LOQ	1.5*LOQ	2*LOQ	LOQ	1.5*LOQ	2*LOQ	LOQ	1.5*LOQ	2*LOQ	LOQ	1.5*LOQ	2*LOQ	LOQ	1.5*LOQ	2*LOQ
		0.780	1.17	1.56	0.780	1.17	1.56	0.780	1.17	1.56	1.56	2.34	3.13	0.640	0.970	1.29
**Intra-day**	Day 1	0.711	1.06	1.65	0.785	1.22	1.56	0.859	1.23	1.63	1.66	2.38	3.17	0.609	0.891	1.44
	Day 2	0.742	1.13	1.56	0.733	1.05	1.48	0.807	1.16	1.59	1.65	2.43	2.90	0.583	0.957	1.36
	Day 3	0.819	1.27	1.74	0.832	1.16	1.45	0.795	1.20	1.51	1.71	2.37	3.09	0.623	0.996	1.28
	Day 4	0.816	1.21	1.60	0.719	1.15	1.40	0.786	1.24	1.54	1.62	2.38	3.02	0.625	0.895	1.19
	Day 5	0.790	1.09	1.45	0.758	1.04	1.59	0.841	1.31	1.65	1.61	2.21	3.22	0.625	0.896	1.35
	Day 6	0.769	1.11	1.38	0.775	1.23	1.42	0.827	1.26	1.50	1.69	2.14	2.83	0.562	0.884	1.28
	%RSD	5.52	6.94	8.37	5.29	7.09	5.06	3.43	4.25	3.75	2.45	4.91	5.00	4.40	4.99	6.55
**Inter-day**	Day 1	0.775	1.14	1.56	0.767	1.10	1.45	0.836	1.28	1.74	1.66	2.32	3.04	0.622	1.03	1.40
	Day 2	0.774	1.13	1.46	0.767	1.14	1.48	0.855	1.27	1.64	1.67	2.13	2.85	0.604	0.920	1.32
	Day 3	0.744	1.05	1.47	0.755	1.08	1.44	0.819	1.23	1.57	1.62	2.17	2.86	0.610	0.891	1.23
	%RSD	2.31	4.35	3.71	0.89	2.87	1.65	2.13	1.85	5.19	1.61	4.45	3.66	1.51	7.59	6.26

For intra-day precision evaluation, six repetitions of each concentration were prepared and injected the same day. For the determination of inter-day precision of the method, six repetitions of each arabidopside concentration were prepared and injected the same day, and the manipulation was repeated three times within the same week.

Signal suppression or enhancement due to matrix effects were evaluated by performing arabidopside dilutions of purified molecules in acetonitrile:water (30:70) in triplicate and by spiking unstressed *Arabidopsis* samples with the purified arabidopsides in triplicate at three different concentration levels (10, 5 and 2.5 µM) inside the calibration range for each molecule. As arabidopsides are highly induced under stress conditions and are only present in low amounts under physiological conditions, arabidopsides were only detected in trace amounts in unstressed plant extracts. The matrix effect (%) corresponded to the peak area in the arabidopside dilution subtracted by the peak area in plant sample, which was then divided by the peak area in the arabidopside dilution and multiplied by 100. Results showed almost no matrix effects for the quantification of the five arabidopside species in plant extracts at the three tested concentrations, showing the good precision of the established method. Calculated matrix effects were respectively between − 0.719% and 4.48% for arabidopside A, − 2.09% and − 0.831% for arabidopside B, 1.88% and 4.30% for arabidopside D, 1.80% and 4.62% for arabidopside E, 2.58% and 6.10% for arabidopside G (Table 3).

**Table 3:** Matrix effects of the established method.

		Arabidopside A			Arabidopside B			Arabidopside D			Arabidopside E			Arabidopside G		
Added concentration (µM)		2.5	5	10	2.5	5	10	2.5	5	10	2.5	5	10	2.5	5	10
Matrix effect (%)	1	3.59	1.66	6.71	− 0.299	− 7.82	− 4.57	− 0.541	2.81	5.79	1.99	− 1.54	− 3.12	5.02	− 5.71	8.23
	2	2.06	− 5.27	− 1.43	1.95	4.36	− 3.46	4.43	0.910	3.10	3.55	5.11	8.24	8.88	7.00	− 0.0468
	3	− 1.57	1.46	8.17	− 4.14	− 2.81	3.87	2.10	1.92	4.01	− 0.134	10.3	5.48	4.40	6.46	2.07
Mean		1.36	− 0.719	4.48	− 0.831	− 2.09	− 1.39	2.00	1.88	4.30	1.80	4.62	3.53	6.10	2.58	3.42
Standard deviation		2.65	3.94	5.17	3.08	6.12	4.59	2.49	0.950	1.37	1.85	5.94	5.92	2.43	7.19	4.30

Matrix effects were evaluated by spiking unstressed plant samples with three different arabidopside concentrations (2.5, 5 and 10 µM). Arabidopside dilutions in acetonitrile:water (30:70) were also realized at the same concentrations. The manipulation was repeated three times with three different unstressed plant samples extracted independently from different *Arabidopsis* plants. Matrix effect (%) corresponded to (arabidopside peak area in the arabidopside dilution subtracted by peak area in the plant sample) divided by peak area in the arabidopside dilution and multiplied by 100.

Recoveries of the extraction method were also determined by analysing two spiked unstressed *Arabidopsis* extracts with the five arabidopsides at 2.5 µM. One sample was directly analysed while the other was resubmitted to the extraction and purification process. The manipulation was performed in triplicate. Acceptable recoveries between 81.5 and 109% were determined for all arabidopsides species, except for arabidopside D, which had recoveries of 70.7%. Calculated recoveries were 81.5% for arabidopside A, 109% for arabidopside B, 70.7% for arabidopside D, 108% for arabidopside E and 89.4% for arabidopside G.

### Method application to plant samples

**Figure 6:**
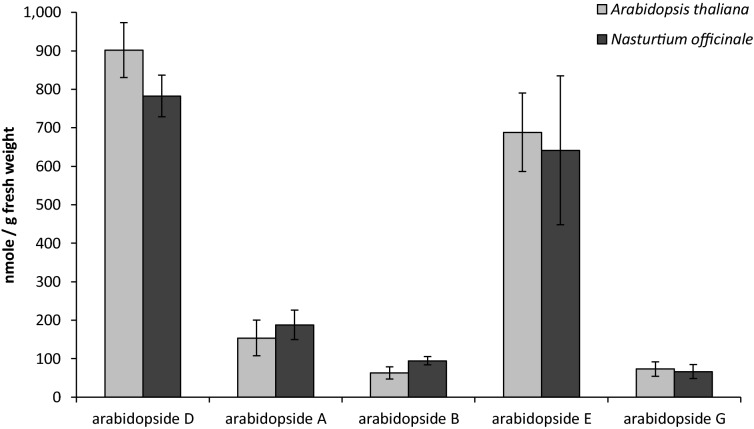
Arabidopside quantification in *Arabidopsis thaliana* and *Nasturtium officinale* 30 min after freeze-thawing (n = 3). Plant lipids were extracted from approximatively 0.4 g of fresh leaves, purified on a SPE silica column and subsequently analysed by HPLC–IT–MS.

The validated method was finally applied to the quantification of the five major arabidopside molecules in two different plant species: *Arabidopsis thaliana* and *Nasturtium officinale*. Results (Fig. 6) showed that arabidopsides D and E are the major species produced by *Arabidopsis* 30 min after freeze-thawing. As arabidopsides A and D have been previously reported in *Nasturtium officinale* leaves^19^, the present method was also applied for the quantification of arabidopsides in that species. Surprisingly, results showed that 30 min after freeze-thawing, arabidopside levels in *Nasturtium officinale* are similar to those of *Arabidopsis thaliana* and that all five major arabidopsides species were produced in concentrations ranging from 63.0 to 902 nmol/g FW. This highlights the importance of those plant metabolites in both plant species, and perhaps in other plants in which arabidopside production has not yet been investigated.

Besides from the detection and quantification of the five major arabidopsides, the described method also allowed detecting other polar lipids in *Arabidopsis* plant extracts. For example, arabidopside C, an oxidized DGDG containing one OPDA and one dnOPDA chain, was also detected with the method as characteristic adduct (959.6, [M + Na]^+^) and fragment (613.4, [M-2 C_6_H_10_O_5_]^+^) were detected for this molecule at a retention time of 14.4 min. However, as this molecule was not purified due to its low level in freeze-thawed *A. thaliana* extracts, its quantification was not performed.

## Conclusion

Arabidopsides are plant metabolites that were first described in *Arabidopsis* but that are now increasingly found in numerous other plant species. Functions of those molecules remain unclear but seem to be crucial for plant defence mechanisms. As low variations in metabolite concentrations can lead to high physiological responses in plants, precise methods are needed for the monitoring of arabidopside formation under specific conditions and in diverse plant species.

Existing methods for arabidopside analysis usually use HPLC–MS/MS or direct infusion MS/MS, with quantification based on an internal calibration with non-oxidized galacto- or phospho-lipids, which largely differ structurally from arabidopsides. As we showed here that even different arabidopside species (oxidized MGDG, oxidized DGDG and oxidized head group acylated MGDG) have distinct analytical responses, there was a high need for new methods allowing precise monitoring of those species in plants. The present HPLC–IT–MS method has been successfully developed and validated for the quantification of five major arabidopsides in plant samples using external calibration with purified authentic arabidopsides. The proposed sample preparation is straightforward, as it requires only extraction, a simple SPE purification procedure and no derivatization before analysis. The high sensitivity, as well as its high accuracy and precision and the low matrix effects, allow the precise quantification of the five arabidopsides in complex plant samples. The present method could be used for the precise monitoring of arabidopside formation under specific conditions, which is key to the understanding of plant defence mechanisms.

## Experimental

### Chemicals and reagents

Ultra-pure deionized water (18 MΩ) was produced using a Q-OPD apparatus. Acetonitrile for LC–MS and glacial acetic acid were purchased from VWR. Methanol, ethyl acetate, isopropanol, chloroform HPLC grade and acetone HPLC grade were obtained from Scharlau. Butan-1-ol, formic acid for LC–MS and ammonium formate for mass spectrometry were purchased from Merck. Heptane was obtained from Biosolve.

### Plant culture

*Arabidopsis thaliana* Columbia 0 (col-0) was cultivated from 6 to 8 weeks with the following photoperiod: 8 h day and 16 h night; as previously described ^9^. The established method was applied to the quantification of arabidopsides in *Arabidopsis* and *Nasturtium officinale* R. Brown. The latter was obtained from the local market.

### Arabidopsides biosynthesis induction, extraction and large scale purification

For large scale purification of arabidopsides, approximatively 100 g of fresh leaves of *A. thaliana* col-0 were frozen in liquid N_2_ for 30 s and left to thaw at room temperature for 30 min as previously described^13^. Thereafter, the leaves were homogenized three times for 10 s using a grinder in cold butan-1-ol:methanol (3:1) with 0.05% (w/v) butylated hydroxytoluene as an antioxidant. The homogenate was agitated for 30 min at 4 °C. After transfer to a separating funnel, phase separation was induced by the addition of 200 mL of heptane:ethyl acetate (3:1) and 200 mL of 2% acetic acid in water. The organic phase was recovered and the aqueous phase re-extracted once again with 300 mL heptane:ethyl acetate (3:1). Organic phases were then pooled and evaporated under vacuum in a rotoevaporator (40 °C). The residue was re-suspended in 25 mL chloroform and kept at − 20 °C before purification.

For that purpose, a silica column Si-60 15–25 µm (4.5 cm height; diameter of 2 cm) was prepared. Undesired apolar lipids were eluted using chloroform:acetone (9:1 v/v) and glycolipids were eluted with acetone:methanol (9:1 v/v). The glycolipid fraction was dried under vacuum in a rotoevaporator at 40 °C, re-suspended in methanol and kept at − 20 °C. Arabidopsides were then purified using preparative HPLC (Puriflash 430, Interchim) with UV detection at 220 nm on a C18 column (uptisphere C18-2 15 µm, 12.2 × 250 mm) (Fig. 2). A gradient of acetonitrile and water was used at a flow of 20 mL min^-1^. After 1 min at 45% acetonitrile, the proportion in acetonitrile was increased to 85% in 17 min and then stabilized for 32 min. Purified arabidopsides were identified by HPLC–MS (Figs. 3 and 4; Supplemental Fig. 1) 2D-NMR (HSQC, COSY, HMBC), UV–visible spectroscopy and infrared spectroscopy (see all spectra for arabidopside A in supplementary data). Arabidopsides were stored at − 20 °C before analysis. Purified arabidopsides were also analysed before and after 2 weeks storage at 25 °C, with no degradation of any of the five molecules.

### HPLC–MS analysis

#### Sample preparation

##### Extraction

Lipids were extracted according to Kourtchenko et al.^9^. Briefly, approximatively 0.4 g of fresh leaves were submerged for 5 min in boiling isopropanol and then dried under a gentle stream of nitrogen. Then, 2 mL of CHCl_3_:methanol:water (1:2:0.8, v:v:v) containing 0.025% of butylated hydroxytoluene were then added, followed by 30 min of sonication and 30 min at 4 °C. Then, 0.5 mL of CHCl_3_ and 0.5 mL of K_2_SO_4_ 380 mM were added to induce phase separation. The organic phase was recovered and the aqueous phase was re-extracted twice with CHCL_3_. All chloroformic extracts were pooled and concentrated under a gentle stream of nitrogen.

##### Clean-up

Raw lipids were dissolved in 500 µL chloroform and separated on a silica column (SiOH, 3 mL/500 mg, Chromabond) before HPLC analysis to improve method specificity and to decrease matrix effects. Volumes of eluting solvents were optimized so that the pre-purification method allows the recovery of all five arabidopsides in the second fraction. Neutral and polar lipids were eluted with 2 mL chloroform:acetone (9:1) and 8 mL acetone:methanol (9:1), respectively. The column was finally washed with 3 mL of methanol to be sure that all molecules of interest were eluted in the previous fraction. Lipid fractions were solubilized in 800 µL acetonitrile:water (30:70 v/v) and diluted 20 times before LC–MS analysis.

#### Chromatographic conditions

Arabidopsides were separated using an HPLC (Agilent 1100) equipped with a C18 column (Inertsil ODS-3 3 µm, 3 × 100 mm, GL Sciences) at a flow of 0.25 mL min^-1^ with a gradient of water (solvent A) and acetonitrile:water (85:15 v/v, solvent B). Solvents contained 0.2% formic acid and 12 mM ammonium formate. The gradient started by a 1-min isocratic elution with 53% B and then linearly programmed to 100% B in 17 min and maintained isocratic for 18 min. The gradient was then reversed in 6 min and stabilized for 3 min. A post time of 2 min was used between each injection. The column temperature was fixed at 40 °C.

#### Mass spectrometric conditions

An Esquire HCT ion trap mass spectrometer (Brüker) was used in electrospray + mode. Mass spectrometer conditions were optimized independently for oxidized MGDGs (arabidopsides A and B), oxidized DGDG (arabidopside D) and oxidized acyl-MGDG (arabidopsides E and G). The capillary voltage was set at − 4500 V, endplate offset at − 500 V, nebulizer pressure at 50 psi and the flow rate of the dry gas at 10 L min^-1^. Mass spectrometer optimized parameters that differed for the analysis of each arabidopside class are presented in Supplemental Table 1. The mass resolution was calculated for each arabidopside as the ration between the mass of the quantification ion and the width of that peak at 50% peak height. Mass resolutions were, respectively, of 1,585 for arabidopside A, 2,051 for arabidopside B, 1,975 for arabidopside D, 2,143 for arabidopside E and 2,199 for arabidopside G. Data treatment was performed using Brüker Daltonics esquire DataAnalysis software.

### Method validation

The method was validated according to European commission recommendation 2002/657/CE. The LOD, LOQ, linearity, precision, accuracy, specificity, matrix effect and recoveries were evaluated. For LOD and LOQ, pure standards of arabidopsides were solubilized in acetonitrile:water (30:70) at different concentrations. The LOD and LOQ corresponded to the concentration giving signal-to-noise ratios of 3 and 10, respectively. Precision (intra-day repeatability and inter-day repeatability, respectively in % RSD_r_ and RSD_R_) and accuracy of the established method were evaluated using dilutions of arabidopsides at three concentrations (LOQ, 1.5*LOQ and 2*LOQ, n = 6). For inter-day repeatability, analyses were repeated three times within the same week. The specificity of the method was assessed by the ability to discriminate arabidopsides from interfering peaks in extracted plant samples. Matrix effects were evaluated by performing arabidopside dilutions in triplicate and by spiking unstressed *Arabidopsis* samples in triplicate at three different arabidopside concentrations (10, 5 and 2.5 µM). Three different unstressed *Arabidopsis* extracts were used as a blank matrix, as arabidopsides are only found in trace amounts in those samples in plant physiological conditions. Matrix effect (%) corresponded to the peak area in the arabidopside dilution subtracted by the peak area in the plant sample, which was then divided by the peak area in the arabidopside dilution and multiplied by 100. Recoveries were also evaluated with unstressed *Arabidopsis* extracts, which went through the whole method process, spiked at one concentration inside the calibration range (2.5 µM). One sample was directly analysed while the other was resubmitted to the whole process before analysis. For each extract, three replicates were performed. Recoveries (%) corresponded to arabidopside peak areas in the extracted sample divided by arabidopside peak area in the non-extracted sample and multiplied by 100.

## Supplementary information

Supplementary Information.

## Data Availability

The datasets generated during and/or analysed during the current study are available from the corresponding author on reasonable request.
