# Supplementary material for: Simple liquid chromatography-electrospray ionization ion trap mass spectrometry method for the quantification of galacto-oxylipin arabidopsides in plant samples
Source: Sci Rep. 2020 Jul 20;10:11957. doi: 10.1038/s41598-020-68757-x (PMC7371884; doi:10.1038/s41598-020-68757-x)
Supplement: Supplementary file 1 — Supplementary Information. [file 41598_2020_68757_MOESM1_ESM.pdf]

Supplementary Information

Simple liquid chromatography - electrospray ionization ion trap mass spectrometry method for the quantification of galacto-oxylipin arabidopsides in plant samples

Manon Genva[a]*, Mats X. Andersson[b] & Marie-Laure Fauconnier[a]

1. HPLC-MS TIC of purified arabidopside standards

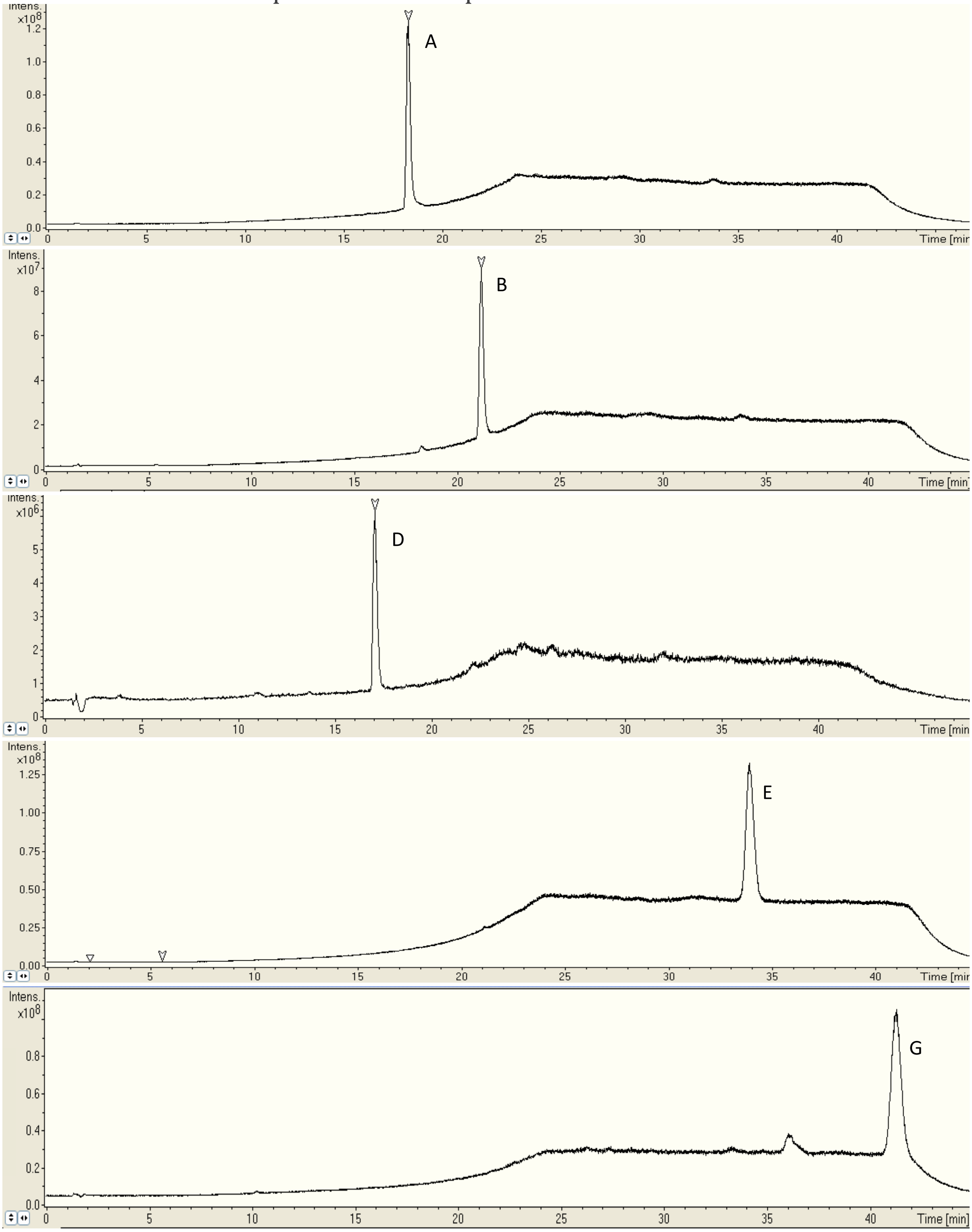

SUPPLEMENTAL FIGURE 1. HPLC-MS TIC OF ARABIDOPSIDE STANDARDS.

2. Arabidopside A spectrometric data

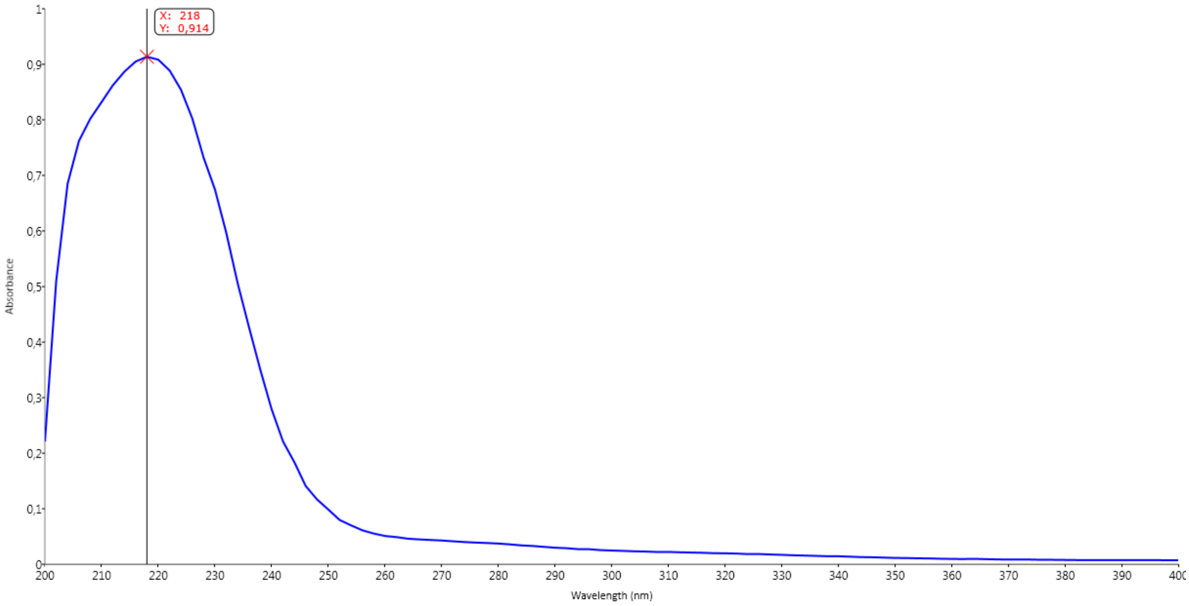

SUPPLEMENTAL FIGURE 2. ARABIDOPSIDE A UV-VISIBLE SPECTRUM.

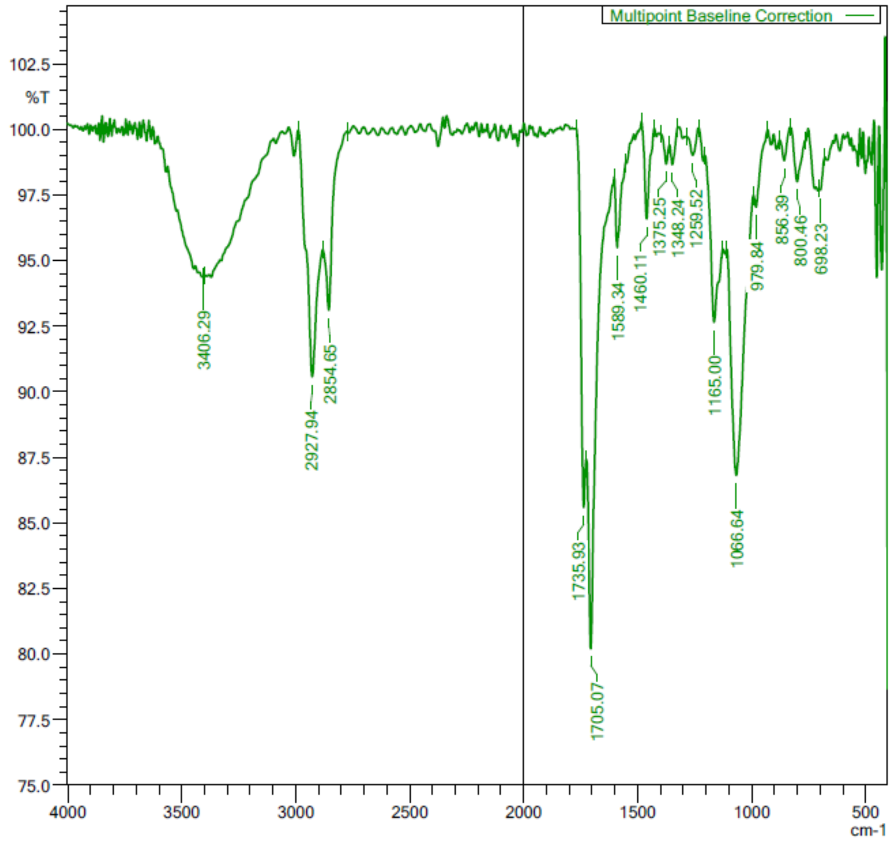

SUPPLEMENTAL FIGURE 3. ARABIDOPSIDE A INFRARED SPECTRUM.

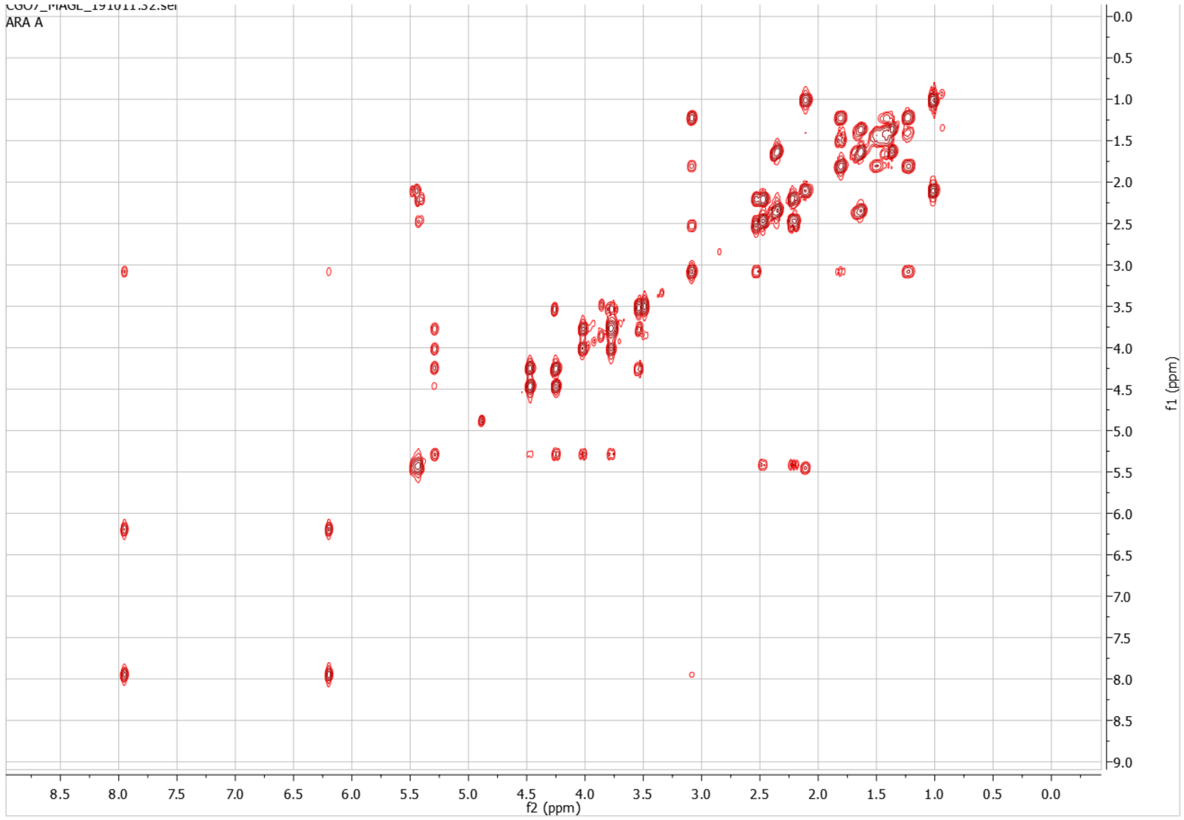

SUPPLEMENTAL FIGURE 4. 2D-COSY ARABIDOPSIDE A SPECTRUM IN MEOD.

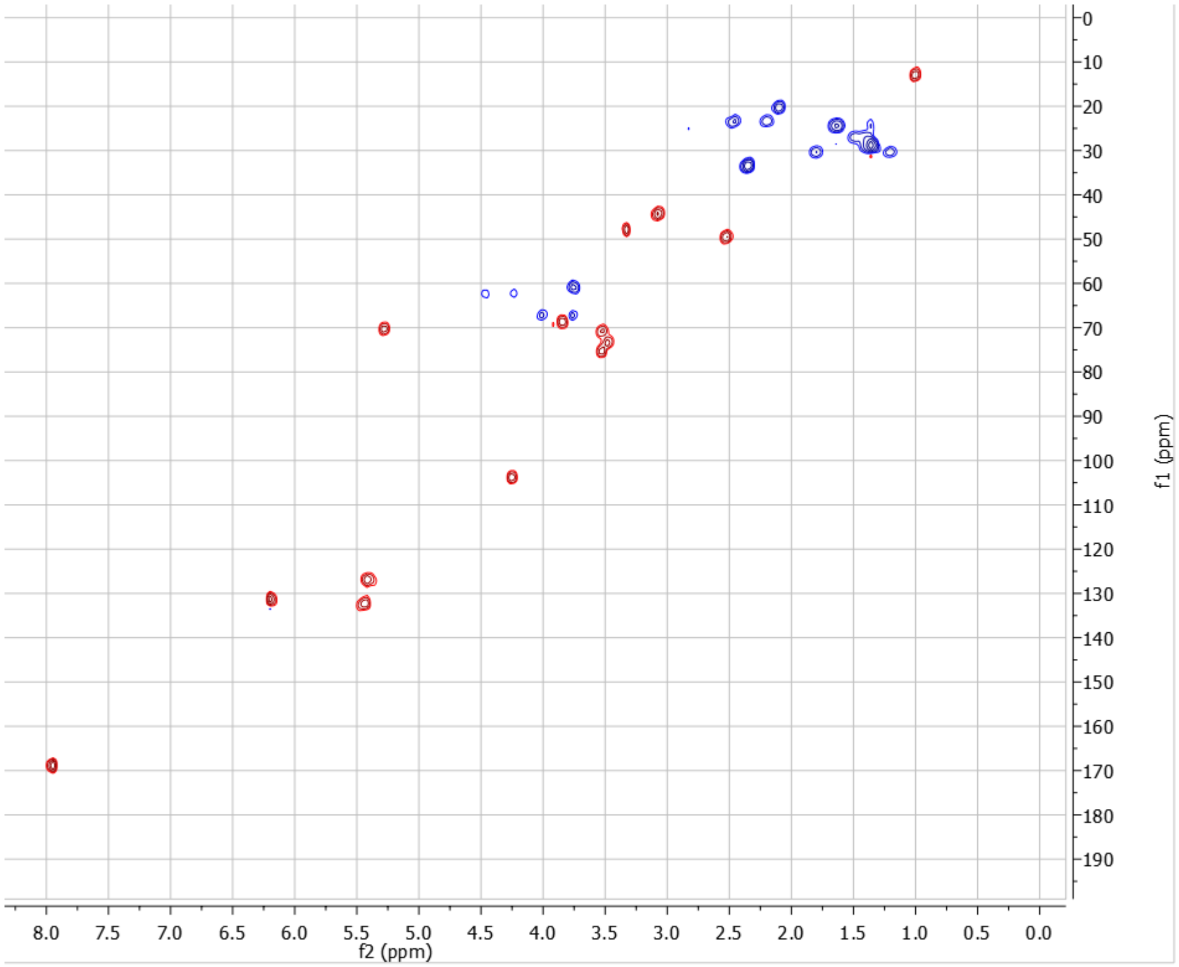

Supplemental Figure 5. 2D-HSQC arabidopside A spectrum in MeOD.

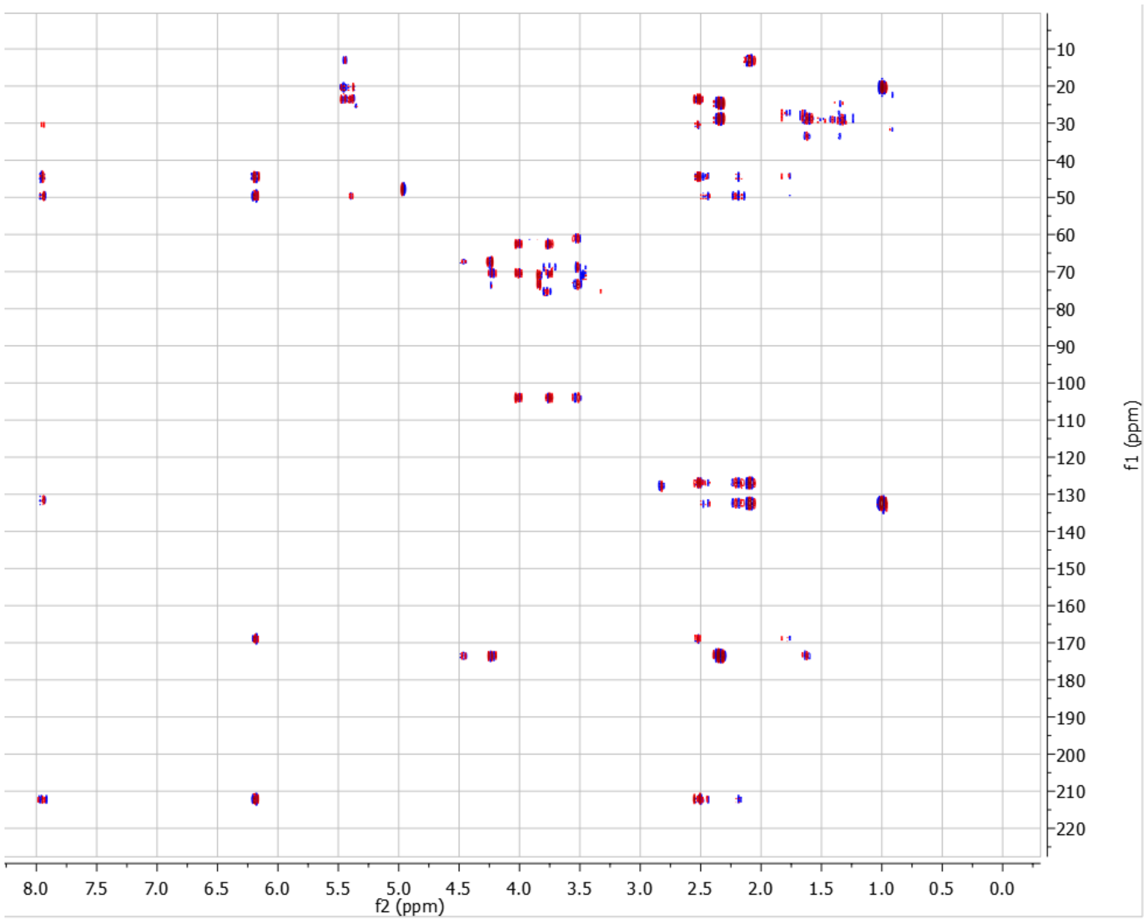

Supplemental Figure 6. 2D-HMBC arabidopside A spectrum in MeOD.

3. HPLC-MS EIC of purified arabidopside standards

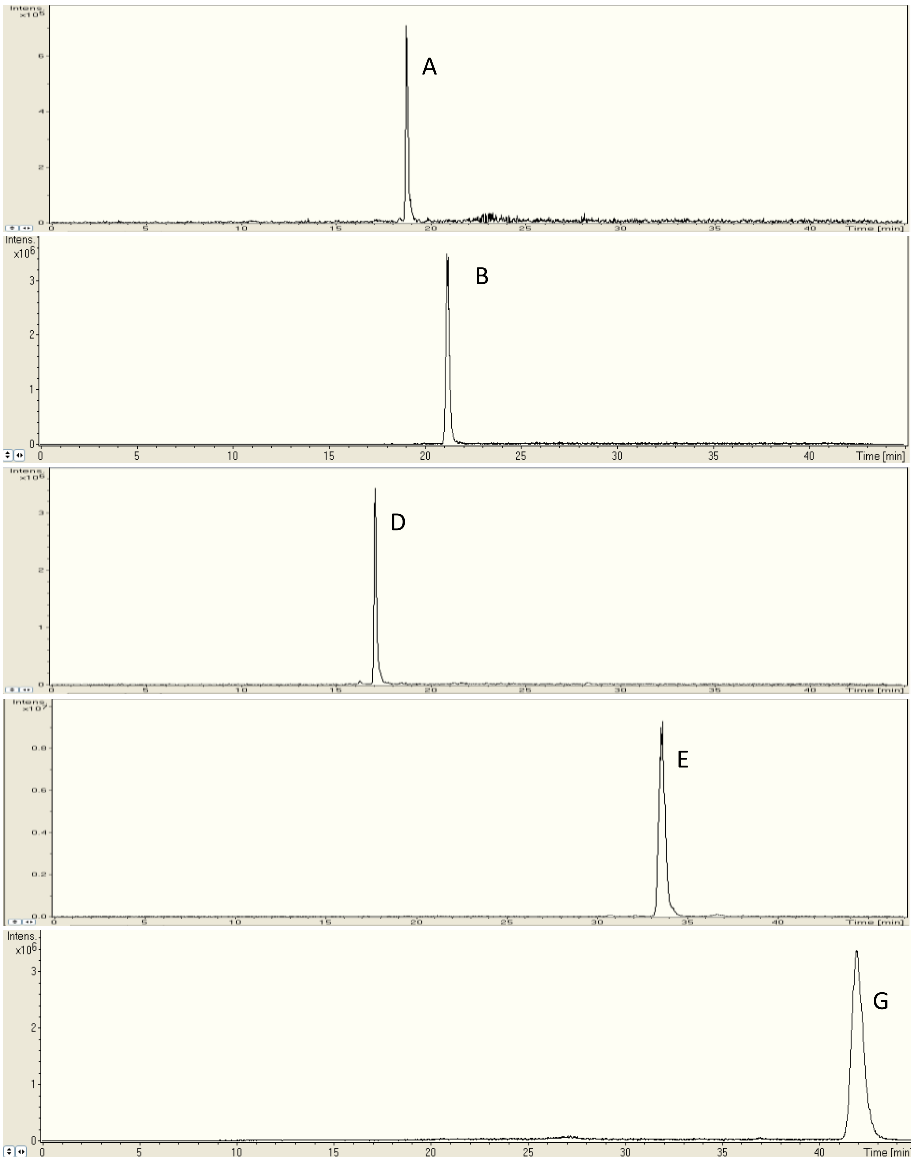

SUPPLEMENTAL FIGURE 7. HPLC-MS EIC OF PURIFIED ARABIDOPSIDE STANDARDS.

4. Arabidopside calibration curves

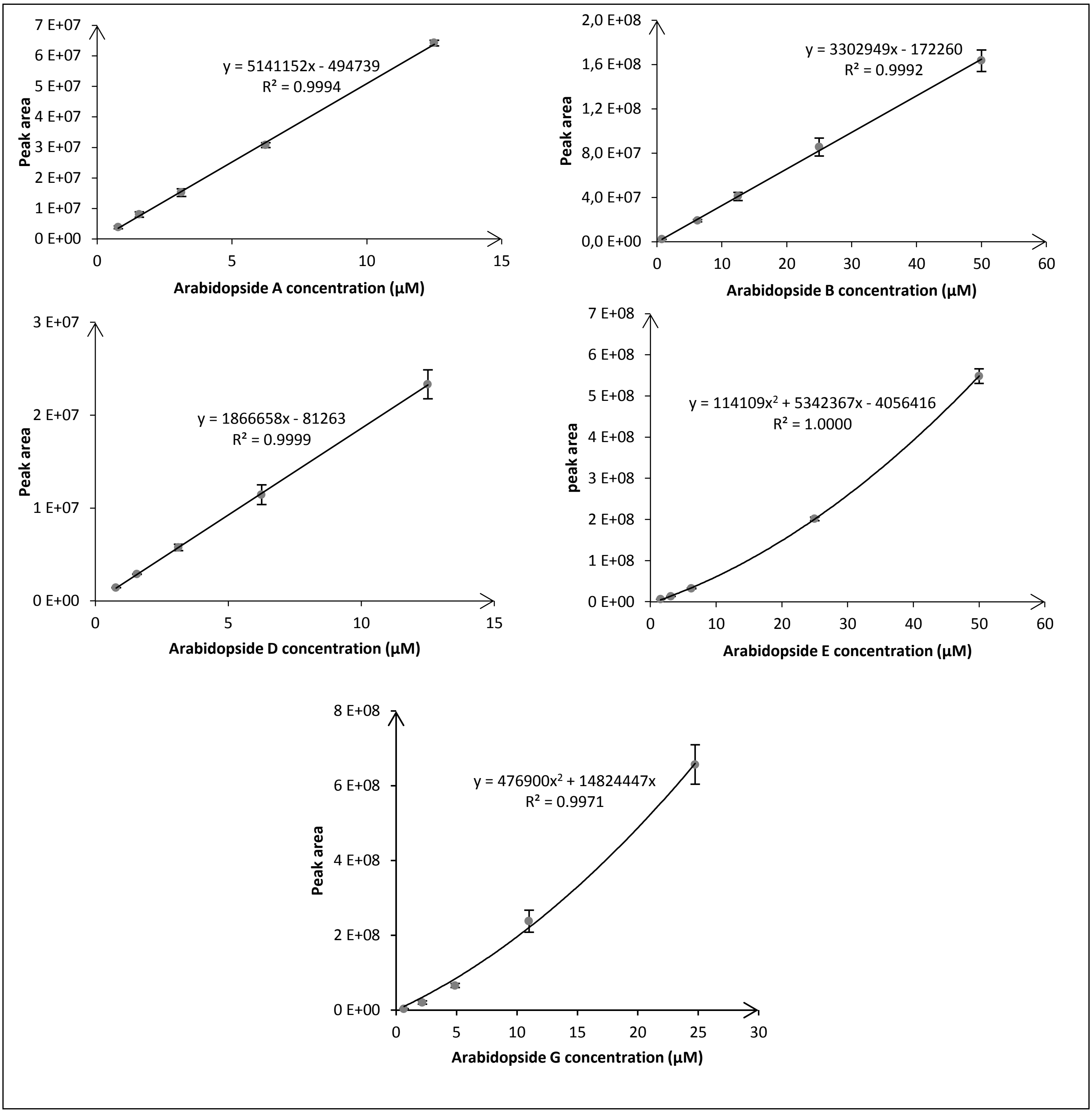

SUPPLEMENTAL FIGURE 8. CALIBRATION CURVES OF ARABIDOPSIDES A, B, D, E AND G USED FOR QUANTIFICATION. EACH CALIBRATION CURVE IS THE MEAN OF THREE DISTINCT CURVES AND EACH POINT CONSISTED IN TRIPLICATE INJECTIONS.

5. Ion trap mass spectrometer parameters for arabidopside quantification

SUPPLEMENTAL TABLE 1. ION TRAP MASS SPECTROMETER OPTIMIZED PARAMETERS FOR THE ANALYSIS OF ARABIDOPSIDES A, B, D, E AND G.

	Arabidopsides A and B	Arabidopside D	Arabidopsides E and G
**Dry Temperature (°C)**	250	300	300
**Skimmer (V)**	38.7	47	20.6
**Cap exit (V)**	150.6	300	300
**Oct 1 DC (V)**	12	9	10
**Oct 2 DC (V)**	2.23	2.75	2.79
**Lens 1 (V)**	-5	-3.7	-5.2
**Lens 2 (V)**	-60	-54.3	-72
**Oct RF (Vpp)**	200	300	300
**Trap drive**	75.6	90	92.8
**Smart target**	150000	200000	200000
**Scan (m/z)**	200 - 1100	200 - 1100	300 - 1250
